# Association of serum total IgE and allergen-specific IgE with insulin resistance in adolescents: an analysis of the NHANES database

**DOI:** 10.1186/s12887-024-04685-3

**Published:** 2024-05-14

**Authors:** Yaping Liu, Xiaoxia Wang, Yong Liu

**Affiliations:** 1 Department of Child Health Care Clinic, Dalian Women and Children’s Medical Group, Dalian, Liaoning 116031 P.R. China; 2 Department of Hematologic Laboratory of Pediatrics, Sun Yat-sen Memorial Hospital, Sun Yat-sen University, No. 107 Yanjiang West Road, Yuexiu District, Guangzhou, Guangdong 510120 P.R. China; 3 Guangdong Provincial Key Laboratory of Malignant Tumor Epigenetics and Gene Regulation, No.107 Yanjiang West Road, Guangzhou, 510120 China

**Keywords:** Total IgE, Allergen-specific IgE, NHANES, Insulin resistance, Adolescents

## Abstract

**Background:**

Recent studies have found that total immunoglobulin E (IgE) and allergen-specific IgE were associated with some metabolic diseases. However, the role of IgE in metabolism among adolescents is still unclear. Herein, this study aims to investigate the associations of serum total IgE and allergen-specific IgE with insulin resistance (IR) in adolescents, in order to provide some reference for the prevention and treatment of metabolic diseases in a young age.

**Methods:**

Data of 870 adolescents were extracted from the National Health and Nutrition Examination Survey (NHANES) database in 2005–2006 in this cross-sectional study. Weighted univariate and multivariate logistic regression analyses were utilized to screen covariates and explore the relationships of serum total IgE and allergen-specific IgE with IR. The evaluation indexes were odds ratios (ORs) and 95% confidence intervals (CIs). In addition, these relationships were also assessed in subgroups of allergy history, asthma history, and number of allergens.

**Results:**

Among eligible adolescents, 168 had IR. No significant association between serum total IgE level and IR was found. However, adolescents with higher level of allergen-specific IgE to rye grass [OR = 0.47, 95%CI: (0.25–0.91)], white oak [OR = 0.57, 95%CI: (0.37–0.88)], or peanut [OR = 0.38, 95%CI: (0.15–0.97)] seemed to have lower odds of IR, whereas those had higher level of shrimp-specific IgE [OR = 2.65, 95%CI: (1.21–5.84)] have increased odds of IR. In addition, these associations between allergen-specific IgE and IR were also discovered in adolescents who had allergy history or asthma history, or had different numbers of allergens.

**Conclusion:**

Paying attention to different allergens in adolescents may be important in the early identification of IR among this high-risk population. The study results relatively provided some reference for further exploration on IR prevention.

**Supplementary Information:**

The online version contains supplementary material available at 10.1186/s12887-024-04685-3.

## Background

Insulin resistance (IR) is a state of responsiveness reduction in insulin-targeting tissues to high physiological insulin levels and considered the pathogenic driver of many diseases [1], which has become an important public health problem in adolescents [2]. The prevalence of IR ranging from 2.1 to 90.8% among adolescents in different stages of sexual maturity and with different nutritional states [3]. Due to IR in adolescents can increase the likelihood of some conditions, such as glucose intolerance, dyslipidemia, endothelial dysfunction, inflammation, and sleep-disordered breathing [4], it is of great necessary to early identify and intervene high-risk populations.

Immunoglobulin E (IgE), a group of immunoglobulins synthesized and released by B lymphocytes, is one of the key components involved in the immune response to allergens. IgE antibodies activates mast cells by binding to fragment crystallized receptors located primarily on the surface of mast cells, and the animal study has shown that mast cells were directly involved in diet-induced obesity and diabetes mellitus (DM) [5]. Total IgE and allergen-specific IgE may be potential risk factors for metabolic diseases and cardiovascular diseases (CVDs). A cross-sectional study among Korean adults suggested that higher serum levels of total IgE, house dust mite IgE, and cockroach IgE were all associated with an increased risk of DM, indicating IgE may be an important independent risk factor for metabolic diseases [6]. Similarly, in the United States, both total IgE levels and allergen-specific IgE levels have been found to be significantly linked to the risk of CVD among adults [7]. In addition, total IgE was also associated with risk of metabolic syndrome (MS) in middle-aged and elderly Chinese persons [8]. IgE as a risk factor is involved in the regulation of atherosclerosis, obesity and IR through regulating macrophage polarization, macrophage-sterol response network gene expression, and foam cell formation [9]. Nevertheless, the roles of total IgE as well as allergen-specific IgE in metabolism related diseases among adolescents have been unclear. Clarify the association of IgE with the risk of IR in adolescents may be beneficial for the exploration on early biomarker to identify high-risk populations and may further reduce the disease burdens.

Herein, this study with the aim of investigating associations of serum total IgE and allergen-specific IgE levels with IR in adolescents, so as to provide some information for further studies on the prevention and treatment of IR at a young age.

## Methods

### Study design and population

Data of adolescents in this cross-sectional study were extracted from the National Health and Nutrition Examination Survey (NHANES) database in 2005–2006. The NHANES is a representative survey research program to assess the health and nutritional status of populations in the United States. Regular data collection is carried out of approximately 5,000 persons from 15 areas since 1999 and examines in two-year periods. The database uses a multi-stage stratified sampling on the basis of selected counties, blocks, households, and persons within households. Data for statistical analyses in this study are collected from the NHANES public use files.

A total of 2,288 adolescents (aged 12–19 years old) were initially included. The exclusion criteria were missing the information on fasting glucose, insulin, serum total IgE concentration, serum allergen-specific IgE concentration, allergy, asthma, height, systolic blood pressure (SBP), energy intake, cotinine, or vitamin D (VD). Finally, 870 were eligible. The NHANES has been approved by the Institutional Review Board (IRB) of the National Center for Health Statistics (NCHS) of the United States Centers for Disease Control and Prevention (CDC). The participation is voluntary and informed consent has been obtained from all participants. Therefore, no ethical approval of our IRB was required since this database was publicly available.

### Measurement of serum total IgE and allergen-specific IgE

In the NHANES, the collected blood samples were used to measure total serum IgE level by the Pharmacia Diagnostics Immuno CAP 1000 System (Pharmacia Diagnostics, Kalamazoo, MI, USA). Details of laboratory and quality control procedures can be obtained on the webpage of the NHANES: https://wwwn.cdc.gov/Nchs/Nhanes/2005-2006/AL_IGE_D.htm. Briefly, anti-IgE covalently coupled to the Immuno Cap reaction vessel reacted with total IgE in the blood sample. Then, enzyme-labeled anti-IgE antibodies were added to form a complex. Using developing agent to incubate the bound complex after washing the unbound enzyme anti-IgE away, and when the reaction was terminated, fluorescence of the eluate was measured. The IgE concentration in the particular sample was proportional to the fluorescence intensity with the lower limit of detection of 2.00 kilounits per liter (kU/L).

According to the previous study, serum total IgE concentration was divided into two levels including < 100 kU/L and ≥ 100 kU/L [10]. We included specific IgE to 19 allergens, including *Dermatophagoides* (*Dermatophagoides farinae*, *Dermatophagoides pteronyssinus*), *Aspergillus* (*Alternaria alternate*, *Aspergillus fumigatus*), botany (common ragweed, rye grass, Bermuda grass, white oak, birch tree, Russian thistle), animals (dog, cat, mouse, rat, German cockroach) and foods (peanut, egg, milk, shrimp). When adolescents had a high level of serum specific IgE to these allergens were recognized as having a positive specific IgE to these allergens (being categorized as “Yes”).

### Definition of insulin resistance

Diagnosis of IR was according to the following formula: homeostatic model assessment (HOMA)-IR = [fasting (> 9-hour fast) glucose (mmol/L) × fasting insulin (µU/mL)] / 22.5 [11]. In our study, IR analyzed as a categorical variable, and the cut-off value was 4.39 units of HOMA-IR, in another word, individuals with HOMA-IR > 4.39 were diagnosed as IR [12].

### Variables selection

We extracted other variables from the NHANES database, including age, gender, race, poverty-to-income ratio (PIR), body mass index (BMI), sedentary time, physical activity, total energy intake, vitamin D (VD), C-reactive protein (CRP), fasting glucose, insulin, cotinine, systolic blood pressure (SBP), diastolic blood pressure (DBP), allergy, asthma, antihypertensive drugs, immunosuppressant, use of immune globulin, and steroids.

We converted adolescents’ BMI to a BMI z-score accounting for age and sex using the recommended CDC percentiles according to the NHANES guidance: https://www.cdc.gov/healthyweight/assessing/bmi/childrens_bmi/about_childrens_bmi.html. A BMI z-score of ≥ 85th percentile indicates an overweight status, and the adolescents were divided into overweight group and non-overweight group. Well-trained and certified inspectors used standardized protocols and calibrated equipment to obtain BP readings. Four consecutive BP readings were obtained through auscultation, and the SBP and DBP was respectively the average of all available measurement data. Non-fasting CRP concentrations were measured using latex-enhanced nephelometry on a Behring Nephelometer.

Physical activity, which was collected through the physical activity questionnaire (PAQ) in the NHANES, was translated into the energy expenditure. Energy expenditure (MET·min) = recommended metabolic equivalent (MET) × exercise time of corresponding activity (min) [13]. Sedentary time (time watching TV/video or using a computer) per average day over the last 30 days was asked in the household interviews. The cut-off points were > 2 h/d for TV, > 1 h/d for computer use, and > 3 h/d for screen time [14].

Information on allergy symptoms was also obtained via the NHANES questionnaires. Participants who had positive answers to one of the following questions were recognized as having allergy symptoms: “During the past 12 months, (have you/has s/he) had a problem with sneezing, or a runny, or blocked nose when (you/s/he) did not have a cold or the flu?” or “Has a doctor or other health professional ever told (you/s/he) that (you have/ s/he has) hay fever?” Asthma was defined by respondents giving a positive response to both questions: “Has a doctor or other health professional ever told you that you have asthma?” and “In the past 12 months (have you/has s/he) had wheezing or whistling in (your/his/her) chest?” [15]. Additionally, drug use status was assessed by the NHANES questionnaires as well as self-reported medical history. More details are available elsewhere: https://wwwn.cdc.gov/Nchs/Nhanes/1999-2000/RXQ_DRUG.htm.

### Statistical analyses

Kolmogorov-Smirnov was used for normality test. Normally distributed data were expressed as mean ± standard error (Mean ± SE), and t test was used for comparison of characteristics between IR and non-IR groups. Non-normally distributed data were represented as median and quartiles [M (Q1, Q3)], and Mann-Whitney U test was used for comparison. Enumeration data were described as frequency and constituent ratio [N (%)] and chi-square test (χ^2^) was used for comparison. Following the NHANES analytical guidelines, special sample weights should be utilized in our research [16]. Due to the test criteria of fasting blood glucose and insulin are very strict, the fasting subsample 2-year MEC weight (WTSAF2YR) should be used in the present study (https://wwwn.cdc.gov/Nchs/Nhanes/2005-2006/GLU_D.htm#SEQN).

Weighted univariate logistic regression analysis was used for covariates screening. Weighted univariate and multivariate logistic regression models were constructed to explore the associations of serum total IgE and allergen-specific IgE with IR in adolescents. The evaluation indexes were odds ratios (ORs) and 95% confidence intervals (CIs). Two-sided *P* < 0.05 is considered significantly associated. Model 1 was the crude model. Model 2 adjusted for variables that significantly associated with IR (*P* < 0.05), including BMI, CRP, SBP, sedentary time, and VD. In addition, due to age [17], gender [18] and race [19] were the factors reported to influence IR, we also included them into the adjustment of multivariate models. Subgroup analyses of allergy history, asthma history, and number of allergens were performed to explore these relationships in different populations. In addition, we investigated the associations of serum total IgE and allergens-specific IgE with IR in adolescents who were allergic to different numbers of allergens to assess whether the associations of different allergens with IR were related to the number of patients with higher IgE classes (the adolescents were divided into number of allergens ≤ 3 group and > 3 group). More details about the basis for grouping were shown in the Table S1. Variables including missing values were deleted or classified into “unknown” category. SAS 9.4 (SAS Institute, Cary, NC, USA) was used for statistical analyses.

## Results

### Characteristics of adolescents

**Fig. 1:**
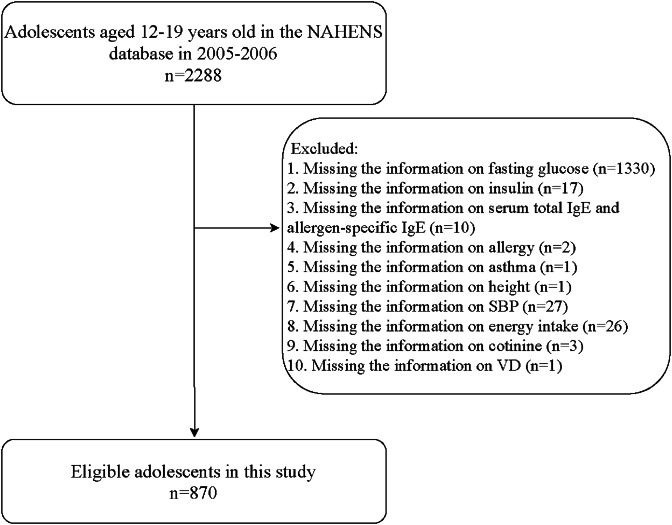
Flowchart of participants screening

Figure 1 is the flowchart of the participants screening. Initially, 2,288 adolescents aged 12–19 years old in the NHANES in 2005–2005 were included. Then those who without information on fasting glucose (*n* = 1330), insulin (*n* = 17), serum total IgE/allergen-specific IgE (*n* = 10), allergy (*n* = 2), asthma (*n* = 1), height (*n* = 1), SBP (*n* = 27), energy intake (*n* = 26), cotinine (*n* = 3) or VD (*n* = 1) were excluded. Finally, 870 were eligible.

Table 1 shows the characteristics of adolescents between IR group and non-IR group. Among eligible adolescents, 168 had IR. The average age of them was 15.51 years old. There were 248 (31.87%) adolescents had allergy, and that 164 (22.36%) had asthma. The average serum total IgE concentrations in IR group and non-IR group were respectively 154.3 kU/L and 178.99 kU/L. The average serum insulin concentration between these two groups was 28.17 uU/mL vs. 8.59 uU/mL. In addition, allergen-specific IgE including *Aspergillus*, *Alternaria alternate*, *Aspergillus fumigatus*, rye grass, birch tree, German cockroach, peanut, and shrimp were significantly different in adolescents between IR group and non-IR group (all *P* < 0.05).

**Table 1:** Characteristic of adolescents in non-IR group and IR group

Variables	Total (*n* = 870)	Non-IR (*n* = 702)	IR (*n* = 168)	Statistics	P
Age, years, Mean (S.E)	15.51 (0.10)	15.58 (0.10)	15.15 (0.27)	t = 1.63	0.124
**Gender, n (%)**				χ^2^ = 1.23	0.267
Male	437 (51.50)	352 (50.70)	85 (55.69)		
Female	433 (48.50)	350 (49.30)	83 (44.31)		
**Race, n (%)**				χ^2^ = 9.96	**0.041**
Mexican American	301 (11.30)	231 (10.45)	70 (15.84)		
Other Hispanic	23 (4.08)	17 (3.20)	6 (8.71)		
Non-Hispanic White	220 (63.94)	186 (65.18)	34 (57.38)		
Non-Hispanic Black	289 (14.40)	238 (14.42)	51 (14.30)		
Other Race - Including Multi-Racial	37 (6.28)	30 (6.76)	7 (3.77)		
**BMI, n (%)**				χ^2^ = 113.12	**< 0.001**
Non-overweight	530 (66.77)	497 (76.38)	33 (16.02)		
Overweight	340 (33.23)	205 (23.62)	135 (83.98)		
CRP, mg/dL, Mean (S.E)	0.20 (0.04)	0.15 (0.01)	0.47 (0.20)	t=-1.57	0.138
SBP, mm Hg, Mean (S.E)	110.43 (1.03)	109.30 (0.83)	116.36 (1.62)	t=-5.81	**< 0.001**
DBP, mm Hg, Mean (S.E)	60.19 (0.88)	60.42 (0.87)	58.92 (1.54)	t = 1.12	0.281
**Sedentary time, hours, n (%)**				χ^2^ = 15.21	**< 0.001**
<3	246 (35.28)	203 (38.24)	43 (19.65)		
3–6	295 (33.08)	235 (32.20)	60 (37.69)		
>6	329 (31.65)	264 (29.56)	65 (42.66)		
VD, nmol/L, Mean (S.E)	62.64 (1.83)	63.57 (1.94)	57.71 (1.95)	t = 2.99	**0.009**
**Physical activity, MET-min/day, n (%)**				χ^2^ = 0.00	0.949
<180	673 (75.60)	544 (75.66)	129 (75.27)		
≥180	197 (24.40)	158 (24.34)	39 (24.73)		
**PIR, n (%)**				χ^2^ = 4.99	0.172
<1.0	254 (18.44)	204 (17.26)	50 (24.66)		
1.0–2.0	203 (17.62)	164 (18.25)	39 (14.28)		
>2.0	371 (60.90)	303 (61.29)	68 (58.85)		
Unknown	42 (3.04)	31 (3.20)	11 (2.22)		
**Allergy, n (%)**				χ^2^ = 1.17	0.280
No	622 (68.13)	503 (66.66)	119 (75.89)		
Yes	248 (31.87)	199 (33.34)	49 (24.11)		
**Asthma, n (%)**				χ^2^ = 0.85	0.356
No	706 (77.64)	577 (78.82)	129 (71.41)		
Yes	164 (22.36)	125 (21.18)	39 (28.59)		
**Antidiabetics, n (%)**				χ^2^ = 1.08	0.298
No	865 (99.36)	699 (99.50)	166 (98.63)		
Yes	5 (0.64)	3 (0.50)	2 (1.37)		
**Antihypertensive drugs, n (%)**					
No	867 (99.53)	702 (100.00)	165 (97.08)		
Yes	3 (0.47)	0 (0.00)	3 (2.92)		
**Immunosuppressant, n (%)**					
No	868 (99.90)	700 (99.88)	168 (100.00)		
Yes	2 (0.10)	2 (0.12)	0 (0.00)		
**Non-immune globulin use, n (%)**					
No	870 (100.00)	702 (100.00)	168 (100.00)		
**Steroid use, n (%)**				χ^2^ = 0.03	0.865
No	833 (93.97)	670 (93.87)	163 (94.46)		
Yes	37 (6.03)	32 (6.13)	5 (5.54)		
Cotinine, mg/dL, Mean (S.E)	24.62 (2.84)	25.66 (3.76)	19.16 (5.86)	t = 0.77	0.453
Energy intake, kcal, Mean (S.E)	2281.58 (52.53)	2293.18 (46.92)	2220.34 (177.60)	t = 0.42	0.683
**Serum total IgE, kU/L, Mean (S.E)**	175.07 (17.97)	178.99 (21.69)	154.38 (20.92)	t = 0.78	0.448
Serum total IgE level, kU/L, n (%)				χ^2^ = 0.94	0.332
<100	523 (63.89)	432 (64.86)	91 (58.79)		
≥100	347 (36.11)	270 (35.14)	77 (41.21)		
**Specific IgE to different allergens**:					
***Dermatophagoides***, n (%)				χ^2^ = 0.44	0.506
No	638 (77.32)	517 (77.85)	121 (74.50)		
Yes	232 (22.68)	185 (22.15)	47 (25.50)		
*Dermatophagoides farinae*, kU/L, Mean (S.E)	4.36 (1.59)	3.96 (1.33)	6.49 (3.06)	t=-1.28	0.220
***Dermatophagoides farinae***, n (%)				χ^2^ = 1.45	0.228
No	665 (79.73)	543 (80.61)	122 (75.08)		
Yes	205 (20.27)	159 (19.39)	46 (24.92)		
*Dermatophagoides pteronyssinus*, kU/L, Mean (S.E)	5.68 (2.51)	5.05 (2.05)	9.00 (4.86)	t=-1.31	0.208
***Dermatophagoides pteronyssinus***, n (%)				χ^2^ = 0.74	0.388
No	654 (78.08)	532 (78.76)	122 (74.50)		
Yes	216 (21.92)	170 (21.24)	46 (25.50)		
***Aspergillus***, n (%)				χ^2^ = 8.04	**0.005**
No	716 (83.88)	578 (82.55)	138 (90.85)		
Yes	154 (16.12)	124 (17.45)	30 (9.15)		
*Alternaria alternate*, kU/L, Mean (S.E)	1.84 (0.39)	2.01 (0.49)	0.92 (0.25)	t = 2.05	0.059
***Alternaria alternate***, n (%)				χ^2^ = 6.33	**0.012**
No	735 (85.51)	592 (84.28)	143 (91.99)		
Yes	135 (14.49)	110 (15.72)	25 (8.01)		
*Aspergillus fumigatus*, kU/L, Mean (S.E)	0.78 (0.16)	0.85 (0.19)	0.38 (0.07)	t = 2.26	**0.039**
***Aspergillus fumigatus***, n (%)				χ^2^ = 4.46	**0.035**
No	753 (89.09)	609 (88.12)	144 (94.20)		
Yes	117 (10.91)	93 (11.88)	24 (5.80)		
**Botany, n (%)**				χ^2^ = 0.86	0.353
No	557 (67.58)	451 (66.81)	106 (71.62)		
Yes	313 (32.42)	251 (33.19)	62 (28.38)		
Common ragweed, kU/L, Mean (S.E)	2.33 (0.78)	2.42 (0.92)	1.88 (0.91)	t = 0.42	0.679
**Common ragweed, n (%)**				χ^2^ = 0.07	0.790
No	664 (77.93)	537 (77.76)	127 (78.83)		
Yes	206 (22.07)	165 (22.24)	41 (21.17)		
Rye grass, kU/L, Mean (S.E)	9.55 (3.10)	10.50 (3.77)	4.50 (1.17)	t = 1.45	0.169
**Rye grass, n (%)**				χ^2^ = 7.17	**0.007**
No	610 (73.05)	489 (71.52)	121 (81.14)		
Yes	260 (26.95)	213 (28.48)	47 (18.86)		
Bermuda grass, kU/L, Mean (S.E)	5.78 (2.25)	6.58 (2.62)	1.53 (0.33)	t = 2.05	0.058
**Bermuda grass, n (%)**				χ^2^ = 3.05	0.081
No	664 (78.10)	532 (77.02)	132 (83.77)		
Yes	206 (21.90)	170 (22.98)	36 (16.23)		
White oak, kU/L, Mean (S.E)	2.04 (0.49)	1.96 (0.46)	2.46 (1.19)	t=-0.45	0.658
**White oak, n (%)**				χ^2^ = 1.24	0.266
No	717 (82.76)	576 (81.98)	141 (86.86)		
Yes	153 (17.24)	126 (18.02)	27 (13.14)		
Birch tree, kU/L, Mean (S.E)	2.59 (0.76)	2.23 (0.50)	4.50 (3.57)	t=-0.64	0.529
**Birch tree, n (%)**				χ^2^ = 3.96	**0.047**
No	734 (84.98)	591 (83.84)	143 (91.01)		
Yes	136 (15.02)	111 (16.16)	25 (8.99)		
Russian thistle, kU/L, Mean (S.E)	1.23 (0.29)	1.35 (0.32)	0.62 (0.21)	t = 2.72	**0.016**
**Russian thistle, n (%)**				χ^2^ = 1.38	0.240
No	713 (84.27)	574 (83.51)	139 (88.30)		
Yes	157 (15.73)	128 (16.49)	29 (11.70)		
**Animals, n (%)**				χ^2^ = 1.43	0.231
No	588 (71.66)	486 (72.69)	102 (66.18)		
Yes	282 (28.34)	216 (27.31)	66 (33.82)		
Cat epithelium and dander, kU/L, Mean (S.E)	1.76 (0.38)	1.52 (0.40)	2.99 (1.38)	t=-1.01	0.328
**Cat epithelium and dander, n (%)**				χ^2^ = 1.21	0.272
No	733 (84.80)	599 (85.35)	134 (81.91)		
Yes	137 (15.20)	103 (14.65)	34 (18.09)		
Dog dander, kU/L, Mean (S.E)	1.04 (0.28)	1.05 (0.32)	0.97 (0.27)	t = 0.19	0.853
**og dander, n (%)**				χ^2^ = 0.16	0.689
No	701 (82.67)	567 (82.41)	134 (84.05)		
Yes	169 (17.33)	135 (17.59)	34 (15.95)		
German cockroach, kU/L, Mean (S.E)	0.72 (0.12)	0.66 (0.13)	1.03 (0.23)	t=-1.67	0.116
**German cockroach, n (%)**				χ^2^ = 7.35	**0.007**
No	715 (86.53)	587 (87.86)	128 (79.51)		
Yes	155 (13.47)	115 (12.14)	40 (20.49)		
Mouse urine proteins, kU/L, Mean (S.E)	0.41 (0.07)	0.39 (0.06)	0.51 (0.20)	t=-0.57	0.580
**Mouse urine proteins, n (%)**				χ^2^ = 1.12	0.290
No	848 (98.69)	686 (98.82)	162 (98.00)		
Yes	22 (1.31)	16 (1.18)	6 (2.00)		
Rat urine proteins, kU/L, Mean (S.E)	0.29 (0.02)	0.28 (0.02)	0.30 (0.04)	t=-0.26	0.797
**Rat urine proteins, n (%)**				χ^2^ = 0.25	0.619
No	853 (98.86)	689 (98.80)	164 (99.16)		
Yes	17 (1.14)	13 (1.20)	4 (0.84)		
**Foods, n (%)**				χ^2^ = 1.12	0.290
No	654 (77.51)	534 (78.49)	120 (72.32)		
Yes	216 (22.49)	168 (21.51)	48 (27.68)		
Peanut, kU/L, Mean (S.E)	0.88 (0.18)	0.92 (0.20)	0.67 (0.24)	t = 0.96	0.354
**Peanut, n (%)**				χ^2^ = 5.18	**0.023**
No	751 (86.24)	603 (85.14)	148 (92.01)		
Yes	119 (13.76)	99 (14.86)	20 (7.99)		
Egg, kU/L, Mean (S.E)	0.26 (0.00)	0.26 (0.00)	0.27 (0.02)	t=-0.63	0.538
**Egg, n (%)**				χ^2^ = 2.54	**0.111**
No	847 (97.05)	684 (96.77)	163 (98.52)		
Yes	23 (2.95)	18 (3.23)	5 (1.48)		
Milk, kU/L, Mean (S.E)	0.28 (0.01)	0.29 (0.01)	0.28 (0.01)	t = 0.70	0.492
**Milk, n (%)**				χ^2^ = 1.74	0.187
No	804 (93.42)	656 (93.94)	148 (90.64)		
Yes	66 (6.58)	46 (6.06)	20 (9.36)		
Shrimp, kU/L, Mean (S.E)	0.48 (0.06)	0.48 (0.07)	0.49 (0.07)	t=-0.06	0.956
**Shrimp, n (%)**				χ^2^ = 8.82	**0.003**
No	791 (93.85)	643 (95.22)	148 (86.63)		
Yes	79 (6.15)	59 (4.78)	20 (13.37)		
Fasting glucose, mmol/L, Mean (S.E)	5.24 (0.05)	5.18 (0.07)	5.54 (0.05)	t=-3.71	**0.002**
Insulin, uU/mL, Mean (S.E)	11.71 (0.60)	8.59 (0.14)	28.17 (1.42)	t=-13.53	**< 0.001**

t: test, χ2: chi-square test

IR: insulin resistance, SE: standard error, BMI: body mass index, CRP: C-reactive protein, SBP: systolic blood pressure, DBP: diastolic blood pressure, VD: vitamin D, MET: metabolic equivalent of energy, PIR: poverty-income ratio, IgE: immunoglobulin E

### Associations of serum total IgE and allergen-specific IgE with IR

Table S2 shows the covariates associated with IR in adolescents. The results showed that BMI, CRP, SBP, sedentary time, and VD were all significantly linked to the odds of IR (all *P* < 0.05).

Then we explored the associations of serum total IgE and allergen-specific IgE, with IR (Table 2). After adjusting for the covariates, no significant relationship between serum total IgE and IR was observed [OR = 1.02, 95%CI: (0.46–2.26)]. However, adolescents had positive allergen-specific IgE, including rye grass [OR = 0.47, 95%CI: (0.25–0.91)], white oak [OR = 0.57, 95%CI: (0.37–0.88)], and peanut [OR = 0.38, 95%CI: (0.15–0.97)], seemed to have lower odds of IR, whereas those had positive shrimp IgE seemed to have higher odds of IR [OR = 2.65, 95%CI: (1.21–5.84)].

**Table 2:** Associations of serum total IgE and allergen-specific IgE with IR in adolescents

Variables	Crude model		Adjusted model	
	OR (95% CI)	P	OR (95% CI)	P
Serum total IgE	1.29 (0.71–2.37)	0.380	1.02 (0.46–2.26)	0.960
**Serum specific IgE to different allergens**				
*Dermatophagoides*	1.20 (0.66–2.19)	0.521	0.83 (0.37–1.86)	0.623
***Dermatophagoides farinae***	1.38 (0.77–2.48)	0.261	1.02 (0.47–2.22)	0.960
*Dermatophagoides pteronyssinus*	1.27 (0.70–2.31)	0.410	0.95 (0.43–2.08)	0.884
***Aspergillus***	0.48 (0.29–0.79)	0.007	0.65 (0.39–1.10)	0.100
*Alternaria alternate*	0.47 (0.26–0.85)	0.016	0.60 (0.32–1.13)	0.106
*Aspergillus fumigatus*	0.46 (0.21–1.01)	0.052	0.79 (0.32–1.97)	0.592
**Botany**	0.80 (0.48–1.33)	0.363	0.65 (0.36–1.15)	0.130
Common ragweed	0.94 (0.57–1.54)	0.791	0.71 (0.38–1.34)	0.273
Rye grass	0.58 (0.38–0.89)	0.015	**0.47 (0.25–0.91)**	**0.029**
Bermuda grass	0.65 (0.39–1.07)	0.086	0.50 (0.25–1.04)	0.061
White oak	0.69 (0.34–1.39)	0.276	**0.57 (0.37–0.88)**	**0.014**
Birch tree	0.51 (0.24–1.07)	0.073	0.51 (0.22–1.15)	0.098
Russian thistle	0.67 (0.33–1.35)	0.245	0.50 (0.22–1.13)	0.088
**Animals**	1.36 (0.76–2.43)	0.275	1.02 (0.47–2.21)	0.957
Cat epithelium and dander	1.29 (0.78–2.13)	0.305	1.13 (0.48–2.65)	0.769
Dog dander	0.89 (0.48–1.66)	0.693	1.07 (0.51–2.25)	0.846
German cockroach	1.86 (1.08–3.21)	0.028	1.25 (0.71–2.22)	0.418
Mouse urine proteins	1.71 (0.58–5.01)	0.304	2.03 (0.55–7.53)	0.268
Rat urine proteins	0.69 (0.14–3.38)	0.631	0.83 (0.24–2.86)	0.752
**Foods**	1.40 (0.72–2.73)	0.303	1.06 (0.42–2.69)	0.889
Peanut	0.50 (0.26–0.97)	0.041	**0.38 (0.15–0.97)**	**0.044**
Egg	0.45 (0.13–1.59)	0.197	0.46 (0.10–2.08)	0.291
Milk	1.60 (0.79–3.25)	0.177	1.64 (0.64–4.22)	0.283
Shrimp	3.07 (1.28–7.40)	0.016	**2.65 (1.21–5.84)**	**0.019**

IgE: immunoglobulin E, IR: insulin resistance, OR: odds ratio, CI: confidence interval

Adjusted model: adjusted for age, gender, race, BMI, CRP, SBP, sedentary time, and VD.

In addition, the serum total IgE concentration and the number of positive allergen-specific IgE in non-IR patients and IR patients were shown in the Table S3. There was no significant difference between these two populations. The Table S4 showed the allergen-specific IgE concentrations between non-IR patients and IR patients respectively, and we only found the Russian thistle allergen-specific IgE (*P* = 0.020) as well as shrimp allergen-specific IgE (*P* < 0.001) were significantly different between non-IR patients and IR patients. These results indicated that the observed associations mentioned above did not due to the IgE levels were higher compared to the other group.

### Relationships of serum total IgE and allergen-specific IgE with IR in different subgroups

Subgroup analyses of allergy history and asthma history were also performed to assess these associations in different populations (Table 3). After adjusting for the covariates, there was still no significantly association between serum total IgE and IR in adolescents (all *P* > 0.05).

**Table 3:** Associations of serum total IgE and allergens-specific IgE with IR in subgroups of allergy and asthma history

Variables	Allergic history		Non-allergic history		Asthma history		Non-asthma history	
	OR (95% CI)	P	OR (95% CI)	P	OR (95% CI)	P	OR (95% CI)	P
Serum total IgE	0.49 (0.13–1.86)	0.272	1.37 (0.60–3.14)	0.433	1.21 (0.23–6.22)	0.811	0.93 (0.36–2.45)	0.883
**Serum specific IgE to different allergens**								
***Dermatophagoides***	0.97 (0.26–3.60)	0.956	0.71 (0.30–1.66)	0.401	2.51 (0.66–9.46)	0.161	0.50 (0.14–1.72)	0.247
*Dermatophagoides farinae*	1.17 (0.37–3.70)	0.775	0.89 (0.37–2.14)	0.774	3.13 (0.75–13.02)	0.109	0.59 (0.17–2.05)	0.385
*Dermatophagoides pteronyssinus*	0.99 (0.26–3.74)	0.981	0.85 (0.36–1.99)	0.686	2.72 (0.69–10.80)	0.142	0.59 (0.16–2.08)	0.383
***Aspergillus***	0.52 (0.19–1.38)	0.175	0.75 (0.25–2.23)	0.582	**0.14 (0.03–0.55)**	**0.008**	0.98 (0.50–1.95)	0.960
*Alternaria alternate*	0.43 (0.15–1.26)	0.114	0.69 (0.18–2.64)	0.562	**0.10 (0.01–0.70)**	**0.023**	0.95 (0.42–2.16)	0.903
*Aspergillus fumigatus*	0.52 (0.14–1.91)	0.302	1.19 (0.27–5.12)	0.807	**0.10 (0.02–0.54)**	**0.011**	1.60 (0.48–5.31)	0.420
**Botany**	**0.23 (0.08–0.72)**	**0.015**	0.85 (0.35–2.06)	0.695	0.48 (0.14–1.66)	0.224	0.57 (0.28–1.18)	0.121
Common ragweed	0.52 (0.20–1.37)	0.172	0.85 (0.42–1.73)	0.628	1.69 (0.26–10.98)	0.557	0.46 (0.14–1.55)	0.192
Rye grass	**0.20 (0.06–0.74)**	**0.019**	0.55 (0.21–1.41)	0.193	0.48 (0.10–2.28)	0.330	**0.34 (0.13–0.94)**	**0.039**
Bermuda grass	**0.21 (0.06–0.77)**	**0.022**	0.61 (0.22–1.69)	0.317	0.76 (0.11–5.15)	0.760	0.31 (0.09–1.07)	0.062
White oak	**0.36 (0.14–0.93)**	**0.037**	0.64 (0.19–2.19)	0.449	0.67 (0.13–3.52)	0.617	**0.40 (0.26–0.61)**	**< 0.001**
Birch tree	**0.34 (0.13–0.92)**	**0.036**	0.44 (0.17–1.13)	0.082	0.69 (0.12–3.90)	0.656	0.27 (0.07–1.01)	0.051
Russian thistle	0.44 (0.14–1.41)	0.154	**0.31 (0.17–0.59)**	**0.001**	1.06 (0.19–5.84)	0.944	**0.24 (0.08–0.74)**	**0.017**
**Animals**	0.53 (0.09–3.12)	0.457	1.18 (0.62–2.23)	0.592	1.73 (0.25–11.91)	0.556	0.81 (0.29–2.22)	0.661
Cat epithelium and dander	0.46 (0.08–2.59)	0.351	1.84 (0.76–4.42)	0.160	1.55 (0.25–9.61)	0.616	0.64 (0.15–2.70)	0.520
Dog dander	0.54 (0.16–1.85)	0.304	1.70 (0.69–4.17)	0.230	2.96 (0.86–10.18)	0.081	0.60 (0.15–2.43)	0.447
German cockroach	2.67 (0.72–9.89)	0.131	1.01 (0.50–2.06)	0.973	**4.62 (1.08–19.80)**	**0.040**	1.09 (0.52–2.28)	0.805
Mouse urine proteins	2.71 (0.47–15.74)	0.246	3.89 (0.19–77.64)	0.349	1.64 (0.30–9.12)	0.546	1.93 (0.03-118.21)	0.737
Rat urine proteins	2.90 (0.75–11.26)	0.116			0.63 (0.02–19.39)	0.779	1.38 (0.12–16.49)	0.784
**Foods**	0.67 (0.16–2.78)	0.558	1.34 (0.55–3.29)	0.498	4.02 (0.73–22.15)	0.103	0.53 (0.19–1.52)	0.219
Peanut	0.34 (0.10–1.17)	0.083	**0.35 (0.16–0.79)**	**0.015**	1.76 (0.18–17.68)	0.609	**0.15 (0.04–0.59)**	**0.010**
Egg	0.93 (0.09–9.84)	0.948	0.55 (0.13–2.32)	0.387			0.16 (0.02–1.37)	0.088
Milk	2.69 (0.71–10.20)	0.135	1.32 (0.24–7.22)	0.734	**4.44 (1.85–10.69)**	**0.003**	1.02 (0.43–2.39)	0.970
Shrimp	2.94 (0.82–10.57)	0.092	3.02 (0.92–9.97)	0.067	4.46 (0.59–33.86)	0.137	1.85 (0.94–3.64)	0.072

IgE: immunoglobulin E, IR: insulin resistance, OR: odds ratio, CI: confidence interval

Adjusted for age, gender, race, BMI, CRP, SBP, sedentary time, and VD.

Adolescents with allergy history who have positive allergen-specific IgE, including rye grass [OR = 0.20, 95%CI: (0.06–0.74)], Bermuda grass [OR = 0.21, 95%CI: (0.06–0.77)], white oak [OR = 0.36, 95%CI: (0.14–0.93)] and birch tree [OR = 0.34, 95%CI: (0.13–0.92)], seemed to have lower odds of IR. In those who without allergy history, having positive allergen-specific IgE of Russian thistle [OR = 0.31, 95%CI: (0.17–0.59)] and peanut [OR = 0.35, 95%CI: (0.16–0.79)] were associated with lower odds of IR.

In asthma subgroup, positive allergen-specific IgE, including *Alternaria alternate* [OR = 0.10, 95%CI: (0.01–0.70)], *Aspergillus fumigatus* [OR = 0.10, 95%CI: (0.02–0.54)] were linked to lower odds of IR, whereas positive allergen-specific IgE of German cockroach [OR = 4.62, 95%CI: (1.08–19.80)] and milk [OR = 4.44, 95%CI: (1.85–10.69)] were associated with higher odds of IR. Adolescents without history of asthma who have allergen-specific IgE including rye grass [OR = 0.34, 95%CI: (0.13–0.94)], white oak [OR = 0.40, 95%CI: (0.26–0.61)], Russian thistle [OR = 0.24, 95%CI: (0.08–0.74)], and peanut [OR = 0.15, 95%CI: (0.04–0.59)] had lower odds of IR.

Moreover, we investigated the associations of total serum IgE and allergen-specific IgE with IR in adolescents who were allergic to different numbers of allergens (Table 4). The results showed that in adolescents are allergic to ≤ 3 allergens, having positive allergen-specific IgE of Rye grass [OR = 0.03, 95%CI: (0.00-0.58)] was associated with lower odds of IR, whereas that of shrimp [OR = 18.85, 95%CI: (1.47-241.51)] was linked to higher odds of IR. Among those who are allergic to more than 3 allergens, positive allergen-specific IgE of white oak [OR = 0.21, 95%CI: (0.06–0.77)] was associated with lower odds of IR, whereas that of egg [OR = 5.36, 95%CI: (1.24–23.15)] was linked to higher odds of IR.

**Table 4:** Associations of serum total IgE and allergens-specific IgE with IR in adolescents who were allergic to different numbers of allergens

Variables	Allergens ≤ 3 *n* = 207		Allergens > 3 *n* = 268	
	OR (95% CI)	P	OR (95% CI)	P
Serum total IgE	2.43 (0.42–14.08)	0.297	1.19 (0.36–3.95)	0.765
**Serum specific IgE to different allergens**				
***Dermatophagoides***	0.35 (0.06–2.01)	0.222	1.49 (0.48–4.60)	0.465
*Dermatophagoides farinae*	0.52 (0.08–3.32)	0.464	1.77 (0.53–5.94)	0.327
*Dermatophagoides pteronyssinus*	0.53 (0.09–3.21)	0.462	1.60 (0.52–4.86)	0.383
***Aspergillus***	0.63 (0.17–2.34)	0.463	0.83 (0.31–2.25)	0.697
*Alternaria alternate*	0.38 (0.06–2.29)	0.268	0.77 (0.24–2.46)	0.632
*Aspergillus fumigatus*	2.01 (0.18–22.63)	0.549	0.62 (0.25–1.55)	0.280
**Botany**	0.80 (0.17–3.67)	0.759	0.12 (0.02–0.75)	**0.027**
Common ragweed	3.43 (0.24–48.39)	0.337	0.41 (0.10–1.66)	0.192
Rye grass	**0.03 (0.00-0.58)**	**0.023**	0.23 (0.05–1.05)	0.057
Bermuda grass	0.22 (0.01–5.06)	0.317	0.24 (0.06–1.03)	0.054
White oak	2.13 (0.04-120.24)	0.695	**0.21 (0.06–0.77)**	**0.022**
Birch tree			0.27 (0.07–1.01)	0.052
Russian thistle	0.24 (0.00-16.03)	0.482	0.30 (0.08–1.23)	0.089
**Animals**	0.79 (0.20–3.07)	0.713	2.99 (0.73–12.28)	0.118
Cat epithelium and dander	0.26 (0.02–2.80)	0.246	1.84 (0.61–5.57)	0.254
Dog dander	0.20 (0.01–7.39)	0.358	1.62 (0.72–3.61)	0.220
German cockroach	1.65 (0.31–8.63)	0.531	1.72 (0.74–3.99)	0.189
Mouse urine proteins			2.08 (0.36–11.91)	0.383
Rat urine proteins			0.65 (0.15–2.85)	0.538
**Foods**	2.30 (0.28–18.76)	0.410	0.78 (0.15–4.20)	0.761
Peanut			0.24 (0.06–1.04)	0.055
Egg			**5.36 (1.24–23.15)**	**0.027**
Milk	2.09 (0.34–12.96)	0.403	1.67 (0.51–5.51)	0.372
Shrimp	**18.85 (1.47-241.51)**	**0.027**	3.29 (0.84–12.86)	0.082

IgE: immunoglobulin E, IR: insulin resistance, OR: odds ratio, CI: confidence interval

Adjusted for age, gender, race, BMI, CRP, SBP, sedentary time, and VD.

## Discussion

Although the relationship between total serum IgE and IR in adolescents was not significant, we observed adolescents have positive allergen-specific IgE of rye grass, white oak, and peanut seemed to have lower odds of IR, whereas that of shrimp seemed to have higher odds. In addition, these relationships of different allergen-specific IgE with IR were quite different in adolescents with/without allergy history or asthma history, as well as those who were allergic to different numbers of allergens.

To the best of our knowledge, this was the first time to explore the associations of serum total IgE and allergen-specific IgE with IR among adolescents. Previous studies focused on the roles of atopic diseases in IR in adolescents, such as asthma, hypersensitivity, and inflammation [20, 21]. No significant association between serum total IgE and IR in adolescents was observed in the current study, and however, previous studies have discussed this relationship. Lee et al. [22] suggested that IR was associated with serum total IgE and atopy in premenopausal women. Zhang et al. [9] showed that IgE deficiency protects mice from IR by regulating macrophage polarization, macrophage-sterol-responsive-network (MSRN) gene expression, and foam cell formation. Differently, Song et al. [23] indicated that asthma-induced high IgE inhibits G6Pase expression in hepatocytes, resulting in the improvement of the IR in liver in male mice. These study results were not consistent, and one possible reason may be attributed to the differences in their study objects [24]. In fact, IR is distinguished by the reduced ability of insulin to stimulate muscle and adipose tissue’s glucose utilization, and suppress hepatic glucose production and output [25, 26]. IR can also cause a resistance to insulin’s function on protein metabolism, lipid metabolism, vascular endothelial function, and gene expression [25, 26]. Allergic sensitization or exacerbation of allergic diseases was associated with interleukin (IL)-6 and activation of toll-like receptors (TLR) 2 and TLR 4, which can promote T helper type 2 (Th2) differentiation, stimulation of these receptors, and subsequent cytokines released, and may further impair glucose homeostasis [27, 28]. IR along with higher level of plasma IL-6 [29], and TLR 2 and TLR 4 are also key mediators of IR [30, 31]. This may be a potential mechanism to explain the role of allergy-related cytokines in the association between allergy and IR. We supposed that the allergen-specific IgE levels that associated with decreased risk of IR among adolescents may because treatment of the allergic reactions could reduce the activation of immune-related receptors and cytokines, which further controlling the course of IR development.

According to our findings, adolescents with positive allergen-specific IgE of rye grass, white oak, and peanut seemed to have lower odds of IR, while that of shrimp seemed to have higher odds. Although no study have respectively explored the association of single allergen-specific IgE with IR in adolescents, these allergens (the most common in the United States) can cause an increase in total serum IgE level [32]. Results from the National Health and Nutrition Examination Survey showed that after the adjustment by all allergens, white oak allergy was independently associated with asthma [33]. Variation in ionized serum calcium (Ca) is associated with the allergic response to common allergens, and bronchial smooth muscle contraction, mast cells granulation and histamine release from mast cells are Ca-dependent [34]. The Ca-binding motifs in allergens, especially of grass origin, are necessary for IgE binding and the chelation of Ca ions from sera of allergic patients lead to decreased allergen-IgE binding or prevented this occurring [35]. Because of asthma was an independent risk factor for IR, this mechanism may explain the potential protective effect of white oak allergy on odds of IR in adolescents. In addition, individuals who were allergic to peanuts had characteristics of oral dysbiosis, reduced oral short chain fatty acid levels, and increased oral mucosal Th2 cytokine secretion [36]. Obesity, including dyslipidemia, plays an important role in the process of IR [21]. However, due to intestinal microbiota is a complicated ecosystem, the association of peanut allergy mediated by abnormal lipid metabolism with IR in adolescents still need further exploration. Oppositely, adolescents who were allergic to shrimp seemed to have high odds of IR. A possible explanation may be that adolescents with seafood allergies, such as shrimp, may not meet healthy dietary pattern requirements, such as the Mediterranean (Med) dietary pattern. Calcaterra et al. [37] suggested that adopt the principle of the Med diet was beneficial to several metabolic derangements including IR in adolescents. Nevertheless, the specific mechanisms that being allergic to rye grass, white oak, or peanut showed a potential protect effect on IR, whereas being allergic to shrimp had the opposite effect are needed to be further clarified.

In adolescents with allergy history or without asthma history, we also found the associations of allergen-specific IgE of rye grass and white oak with IR. The relationship between positive allergen-specific IgE of peanut and lower odds of IR was observed in those who not have the history of allergy or asthma. Also, the positive allergen-specific IgE of shrimp was linked to higher odds of IR in adolescents who are allergic to ≤ 3 allergens. In addition, not difficult to find that adolescents had allergy history especially the botany allergy, including rye grass, Bermuda grass, white oak, and birch tree, as well as had asthma history especially the *Aspergillums* allergy, seemed to have lower odds of IR. These relationships are consistent with the underlying mechanisms we speculated above. Interestingly, being allergic to German cockroach or milk was associated with higher odds of IR in adolescents had asthma history. Study in Korean adults found that subjects sensitized to the German cockroach were at increased risk of DM [6]. Protease-activated receptor-2 (PAR-2) activation has been implicated in the potent allergenicity elicited by cockroaches and contributes substantially to inflammatory and metabolic dysfunction [38], and PAR-2 antagonists inhibit diet-induced obesity, reverse IR, and glucose intolerance, and they beneficially modulate liver and pancreatic metabolic parameters [39]. Our results indicated that elevated levels of German cockroach specific IgE may promote the development of inflammation, possibly exacerbating airway hyperresponsiveness on asthma adolescents and bring about undesirable consequences. Similarly, higher milk specific IgE level was also related to the higher odds of IR. Gong et al. [40] suggested that specific bioactive peptides from goat milk casein hydrolysates ameliorated insulin resistance in HepG2 cells that had been treated with high glucose. Studies by Delgadillo-Puga et al. [41] and Chakrabarti et al. [42] also discovered the same phenomenon. These results prompted that allergic to milk may associated with IR. Subgroup analysis results indicated that adolescents with/without history of allergy or asthma should focus on the allergen monitoring, adopting the principle of the Med diet, and keeping appropriate physical activities in daily life, which may help reduce the risk of IR.

The current study basing on the NHANES database explored the roles of serum total IgE and allergen-specific IgE in IR among adolescents, which relatively filled the literature gap in this field. However, there are still some limitations. As a cross-sectional study, it could not infer the causal associations of serum total IgE and allergen-specific IgE with IR. The NHANES only detects the expression of 19 common allergen-specific IgE in blood samples, and although other potential related allergens are omitted, it already contains common types in daily life. This study was limited to only 1 two-year cycle data in the database, and there were very few positive samples for some allergen-specific IgE such as rat-specific IgE level that was difficult to output the results. Therefore, it is necessary to conduct larger, more complete prospective cohort studies to confirm the associations of serum total IgE and allergic-specific IgE with IR among adolescents in the future.

## Conclusion

Allergen-specific IgE levels were associated with the odds of IR in adolescents. Paying attention to adolescents who are allergic to different allergens may be important in the early identification of this high IR risk population.

### Electronic supplementary material

Below is the link to the electronic supplementary material.

Supplementary Material 1

## Abbreviations

IR: Insulin resistance
IgE: Immunoglobulin E
NHANES: National Health and Nutrition Examination Survey
IRB: Institutional Review Board
NCHS: National Center for Health Statistics
CDC: Centers for Disease Control and Prevention
HOMA-IR: Homeostatic model assessment of insulin resistance
PIR: poverty-to-income ratio
BMI: body mass index
SBP: systolic blood pressure
DBP: diastolic blood pressure
MET: Metabolic equivalent
IQR: interquartile range
ORs: odds ratios
