# Supplementary material for: Association of serum total IgE and allergen-specific IgE with insulin resistance in adolescents: an analysis of the NHANES database
Source: BMC Pediatr. 2024 May 14;24:332. doi: 10.1186/s12887-024-04685-3 (PMC11092217; doi:10.1186/s12887-024-04685-3)
Supplement: Supplementary file 1 — Supplementary Material 1 [file 12887_2024_4685_MOESM1_ESM.docx]

**Table S1 Numbers of the allergens in non-IR group and IR group**

| Variables | Total  (n=475) | Non-IR (n=377) | IR  (n=98) | Statistics | *P* |
| --- | --- | --- | --- | --- | --- |
| Total number of allergen-specific IgE, Mean (S.E) | 5.48 (0.31) | 5.60 (0.37) | 4.91 (0.45) | t=1.12 | 0.281 |
| Different numbers of positive allergen-specific IgE, n (%) |  |  |  |  |  |
| 1 | 97 (18.18) | 74 (16.37) | 23 (27.44) |  |  |
| 2 | 66 (16.42) | 55 (18.52) | 11 (5.73) |  |  |
| 3 | 44 (9.57) | 40 (10.29) | 4 (5.90) |  |  |
| 4 | 47 (9.96) | 32 (8.10) | 15 (19.48) |  |  |
| 5 | 20 (3.70) | 18 (3.40) | 2 (5.24) |  |  |
| 6 | 25 (3.71) | 17 (3.01) | 8 (7.25) |  |  |
| 7 | 30 (5.70) | 21 (5.23) | 9 (8.05) |  |  |
| 8 | 25 (5.14) | 20 (5.77) | 5 (1.93) |  |  |
| 9 | 29 (5.98) | 25 (6.09) | 4 (5.43) |  |  |
| 10 | 23 (5.55) | 19 (6.41) | 4 (1.21) |  |  |
| 11 | 21 (6.95) | 18 (7.36) | 3 (4.88) |  |  |
| 12 | 17 (3.04) | 15 (2.95) | 2 (3.48) |  |  |
| 13 | 11 (2.76) | 9 (3.22) | 2 (0.44) |  |  |
| 14 | 10 (1.72) | 8 (1.74) | 2 (1.64) |  |  |
| 15 | 6 (1.38) | 3 (1.35) | 3 (1.50) |  |  |
| 17 | 3 (0.18) | 3 (0.21) | 0 (0.00) |  |  |
| 19 | 1 (0.06) | 0 (0.00) | 1 (0.38) |  |  |
| Total number of positive allergen-specific IgE, n (%) |  |  |  | χ^2^=1.152 | 0.283 |
| ≤3 | 207 (44.17) | 169 (45.17) | 38 (39.07) |  |  |
| >3 | 268 (55.83) | 208 (54.83) | 60 (60.93) |  |  |

IR: insulin resistance, IgE: immunoglobulin E, SE: standard error.

**Table S2 Covariates associated with IR in adolescents**

| Variables | OR (95% CI) | *P* |
| --- | --- | --- |
| Age | 0.92 (0.83-1.03) | 0.130 |
| Gender |  |  |
| Male | Ref |  |
| Female | 0.82 (0.55-1.21) | 0.292 |
| Race |  |  |
| Mexican American | Ref |  |
| Other Hispanic | 1.79 (0.44-7.29) | 0.389 |
| Non-Hispanic White | 0.58 (0.32-1.04) | 0.066 |
| Non-Hispanic Black | 0.65 (0.40-1.06) | 0.083 |
| Other Race - Including Multi-Racial | 0.37 (0.13-1.01) | 0.052 |
| BMI |  |  |
| Non-overweight | Ref |  |
| Overweight | 16.95 (7.24-39.68) | **<0.001** |
| CRP | 1.67 (1.17-2.38) | **0.008** |
| SBP | 1.07 (1.04-1.11) | **<0.001** |
| DBP | 0.99 (0.97-1.01) | 0.287 |
| Sedentary time |  |  |
| <3 | Ref |  |
| 3-6 | 2.28 (1.11-4.69) | **0.028** |
| >6 | 2.81 (1.46-5.41) | **0.004** |
| VD | 0.99 (0.97-0.99) | **0.022** |
| Physical activity |  |  |
| <180 | Ref |  |
| ≥180 | 1.02 (0.51-2.04) | 0.949 |
| PIR |  |  |
| <1.0 | Ref |  |
| 1.0-2.0 | 0.55 (0.26-1.16) | 0.109 |
| >2.0 | 0.67 (0.41-1.09) | 0.101 |
| Unknown | 0.49 (0.19-1.26) | 0.127 |
| Allergy |  |  |
| No | Ref |  |
| Yes | 0.64(0.26-1.56) | 0.300 |
| Asthma |  |  |
| No | Ref |  |
| Yes | 1.49(0.59-3.76) | 0.372 |
| Antidiabetic |  |  |
| No | Ref |  |
| Yes | 2.77 (0.32-24.11) | 0.332 |
| Steroid |  |  |
| No | Ref |  |
| Yes | 0.90 (0.23-3.45) | 0.866 |
| Cotinine | 1.00 (0.99-1.00) | 0.479 |
| Energy intake | 1.00 (1.00-1.00) | 0.695 |

IR: insulin resistance, OR: odds ratio, CI: confidence interval, Ref: reference, BMI: body mass index, CRP: C-reactive protein, SBP: systolic blood pressure, DBP: diastolic blood pressure, VD: vitamin D, PIR: poverty-income ratio

**Table S3 Serum total IgE and allergen-specific IgE level among different populations**

| Variables | Total IgE level ≥100 kU/L  (n=347) | Non-IR (n=270) | IR  (n=77) | Statistics | *P* |
| --- | --- | --- | --- | --- | --- |
| Serum total IgE antibody, kU/L, Mean (S.E) | 430.03 (38.21) | 452.99 (41.63) | 326.73 (52.29) | t=1.96 | 0.069 |
| Number of positive allergen-specific IgE, Mean (S.E) | 5.91 (0.39) | 6.14 (0.42) | 4.87 (0.61) | t=1.79 | 0.094 |

IgE: immunoglobulin E, IR: insulin resistance, SE: standard error.

**Table S4 Serum allergen-specific IgE concentrations between non-IR persons and IR patients**

| Allergen | Total  (n=205) | Non-IR (n=159) | IR  (n=46) | Statistics | *P* |
| --- | --- | --- | --- | --- | --- |
| *Dermatophagoides farinae*, Mean (S.E) | 20.52 (8.15) | 19.36 (7.02) | 25.28 (13.57) | t=-0.81 | 0.430 |
| Allergen | Total  (n=216) | Non-IR (n=170) | IR  (n=46) | Statistics | *P* |
| *Dermatophagoides pteronyssinus*, Mean (S.E) | 25.03 (11.64) | 22.86 (9.68) | 34.57 (20.93) | t=-1.00 | 0.334 |
| Allergen | Total  (n=135) | Non-IR (n=110) | IR  (n=25) | Statistics | *P* |
| *Alternaria alternate*, Mean (S.E) | 11.23 (2.01) | 11.48 (2.29) | 8.64 (3.34) | t=0.63 | 0.535 |
| Allergen | Total  (n=117) | Non-IR (n=93) | IR  (n=24) | Statistics | *P* |
| *Aspergillus fumigatus*, Mean (S.E) | 5.08 (1.21) | 5.32 (1.30) | 2.50 (1.43) | t=1.48 | 0.160 |
| Allergen | Total  (n=206) | Non-IR (n=165) | IR  (n=41) | Statistics | *P* |
| Common ragweed, Mean (S.E) | 9.69 (3.09) | 10.00 (3.70) | 7.94 (4.52) | t=0.33 | 0.743 |
| Allergen | Total  (n=260) | Non-IR (n=213) | IR  (n=47) | Statistics | *P* |
| Rye grass, Mean (S.E) | 34.74 (12.30) | 36.24 (13.94) | 22.79 (5.83) | t=0.87 | 0.397 |
| Allergen | Total  (n=206) | Non-IR (n=170) | IR  (n=36) | Statistics | *P* |
| Bermuda grass, Mean (S.E) | 25.48 (10.42) | 27.80 (11.56) | 8.13 (2.40) | t=1.93 | 0.073 |
| Allergen | Total  (n=153) | Non-IR (n=126) | IR  (n=27) | Statistics | *P* |
| White oak, Mean (S.E) | 10.65 (2.98) | 9.76 (2.50) | 17.10 (10.29) | t=-0.79 | 0.443 |
| Allergen | Total  (n=136) | Non-IR (n=111) | IR  (n=25) | Statistics | *P* |
| Birch tree, Mean (S.E) | 15.85 (5.35) | 12.51 (3.27) | 47.47 (40.99) | t=-0.86 | 0.402 |
| Allergen | Total  (n=157) | Non-IR (n=128) | IR  (n=29) | Statistics | *P* |
| Russian thistle, Mean (S.E) | 6.49 (1.46) | 6.90 (1.43) | 3.43 (1.85) | t=2.61 | **0.020** |
| Allergen | Total  (n=137) | Non-IR (n=103) | IR  (n=34) | Statistics | *P* |
| Cat epithelium and dander, Mean (S.E) | 10.17 (2.95) | 8.95 (2.93) | 15.41 (7.10) | t=-0.92 | 0.371 |
| Allergen | Total  (n=169) | Non-IR (n=135) | IR  (n=34) | Statistics | *P* |
| Dog dander, Mean (S.E) | 4.81 (1.67) | 4.81 (1.96) | 4.79 (1.74) | t=0.01 | 0.996 |
| Allergen | Total  (n=155) | Non-IR (n=115) | IR  (n=40) | Statistics | *P* |
| German cockroach, Mean (S.E) | 3.72 (0.96) | 3.60 (1.04) | 4.07 (1.46) | t=-0.31 | 0.760 |
| Allergen | Total  (n=22) | Non-IR (n=16) | IR  (n=6) | Statistics | *P* |
| Mouse urine proteins, Mean (S.E) | 12.58 (3.01) | 12.45 (3.03) | 12.99 (10.10) | t=-0.04 | 0.966 |
| Allergen | Total  (n=17) | Non-IR (n=13) | IR  (n=4) | Statistics | *P* |
| Rat urine proteins, Mean (S.E) | 3.41 (0.62) | 3.11 (0.75) | 5.71 (0.95) | t=-0.57 | 0.584 |
| Allergen | Total  (n=119) | Non-IR (n=99) | IR  (n=20) | Statistics | *P* |
| Peanut, Mean (S.E) | 4.82 (1.12) | 4.76 (1.07) | 5.49 (3.31) | t=-0.25 | 0.806 |
| Allergen | Total  (n=23) | Non-IR (n=18) | IR  (n=5) | Statistics | *P* |
| Egg, Mean (S.E) | 0.65 (0.12) | 0.55 (0.03) | 1.72 (1.43) | t=-0.82 | 0.430 |
| Allergen | Total  (n=66) | Non-IR (n=46) | IR  (n=20) | Statistics | *P* |
| Milk, Mean (S.E) | 0.76 (0.12) | 0.84 (0.15) | 0.52 (0.06) | t=1.85 | 0.087 |
| Allergen | Total  (n=79) | Non-IR (n=59) | IR  (n=20) | Statistics | *P* |
| Shrimp, Mean (S.E) | 4.02 (0.99) | 5.08 (1.32) | 2.01 (0.62) | t=Infty | **<0.001** |

*Each total population was those who had allergen-specific IgE concentrations >0.35 kU/L.

IgE: immunoglobulin E, IR: insulin resistance, SE: standard error.
